# Genomic insights into bacteriophages: a new frontier in AMR detection and phage therapy

**DOI:** 10.1093/bfgp/elaf011

**Published:** 2025-07-28

**Authors:** Basudha Banerjee, Sayanti Halder, Shubham Kumar, Muskan Chaddha, Raiyan Ali, Ramakant Mohite, Muskan Bano, Rajesh Pandey

**Affiliations:** Division of Immunology and Infectious Disease Biology, INtegrative GENomics of Hope-PathogEn (INGEN-HOPE) Laboratory, CSIR–Institute of Genomics and Integrative Biology (CSIR-IGIB), Mall Road, Delhi 110007, India; Division of Immunology and Infectious Disease Biology, INtegrative GENomics of Hope-PathogEn (INGEN-HOPE) Laboratory, CSIR–Institute of Genomics and Integrative Biology (CSIR-IGIB), Mall Road, Delhi 110007, India; Division of Immunology and Infectious Disease Biology, INtegrative GENomics of Hope-PathogEn (INGEN-HOPE) Laboratory, CSIR–Institute of Genomics and Integrative Biology (CSIR-IGIB), Mall Road, Delhi 110007, India; Division of Immunology and Infectious Disease Biology, INtegrative GENomics of Hope-PathogEn (INGEN-HOPE) Laboratory, CSIR–Institute of Genomics and Integrative Biology (CSIR-IGIB), Mall Road, Delhi 110007, India; Division of Immunology and Infectious Disease Biology, INtegrative GENomics of Hope-PathogEn (INGEN-HOPE) Laboratory, CSIR–Institute of Genomics and Integrative Biology (CSIR-IGIB), Mall Road, Delhi 110007, India; Division of Immunology and Infectious Disease Biology, INtegrative GENomics of Hope-PathogEn (INGEN-HOPE) Laboratory, CSIR–Institute of Genomics and Integrative Biology (CSIR-IGIB), Mall Road, Delhi 110007, India; Division of Immunology and Infectious Disease Biology, INtegrative GENomics of Hope-PathogEn (INGEN-HOPE) Laboratory, CSIR–Institute of Genomics and Integrative Biology (CSIR-IGIB), Mall Road, Delhi 110007, India; Division of Immunology and Infectious Disease Biology, INtegrative GENomics of Hope-PathogEn (INGEN-HOPE) Laboratory, CSIR–Institute of Genomics and Integrative Biology (CSIR-IGIB), Mall Road, Delhi 110007, India; Academy of Scientific and Innovative Research (AcSIR), Ghaziabad 201002, India

**Keywords:** bacteriophages, AMR, genomics, Holo-transcriptomics, phage therapy, disease severity

## Abstract

The misuse and overprescription of antibiotics have accelerated the rise of antimicrobial resistance (AMR), rendering many antibiotics ineffective and leading to significant clinical challenges. The conventional treatment methods have become progressively challenging, posing a threat of evolving into an impending silent pandemic. The long track record of bacteriophages combating bacterial infections has renewed hope into the potential therapeutic benefits of bacteriophages. Bacteriophage therapy offers a promising alternative to antibiotics, particularly against multidrug-resistant (MDR) pathogens. This article explores the promise of phages as a potential means to combat superbugs from the perspective of the genomic and transcriptomic landscape of the phages and their bacterial host. Advances in bacteriophage genomics have expedited the detection of new phages and AMR genes, enhancing our understanding of phage-host interactions and enabling the identification of potential treatments for antibiotic-resistant bacteria. At the same time, holo-transcriptomic studies hold potential for discovering disease and context-specific transcriptionally active phages vis-à-vis disease severity. Holo-transcriptomic profiling can be applied to investigate the presence of AMR-bacteria, highlighting COVID-19 and Dengue diseases, in addition to the globally recognized ESKAPE pathogens. By simultaneously capturing phage, bacterial and host transcripts, this approach enables a better comprehension of the bacteriophage dynamics. Moreover, insight into these defence and counter–defence interactions is essential for augmenting the adoption of phage therapy at scale and advancing bacterial control in clinical settings.

## Introduction

Antibiotics have been the foundation of medicine for a century; however, bacterial infections have grown to create significant casualties globally. Excessive use of antibiotics has led to widespread antimicrobial resistance (AMR). During AMR, bacteria acquire properties that make antibiotics less effective. This alarming crisis in bacterial pathogens makes fighting infectious diseases a herculean task [1]. In 2021, ~4.71 million deaths were reported to be associated with AMR. Bacterial AMR severely impacts patient recovery, significantly contributing to higher mortality rates [2]. From 1990 to 2021, it surged by 80% in adults aged 70 years and above [2]. An estimate of 10 million deaths in a year has been predicted by 2050 [3]. WHO has also declared AMR to be the top global public health threat [4]. Given the growing ineffectiveness of antibiotics, alternative therapeutic approaches need to be urgently explored. With this notion, bacteriophages—viruses capable of infecting and invading bacterial cells are being investigated anew as an antimicrobial therapy where they might be used alone, as a phage cocktail, or in synergy with antibiotics [5]. One of the most striking and defining aspects of the bacteriophage genome is its mosaicism. The mosaic nature of the genome occurs due to discrete evolutionary histories and horizontal gene transfer (HGT) after acquiring genetic sequences of the bacterial host [6]. This genomic variability raises concerns about the unintended spread of resistance genes [7, 8]. Bacterial resistance mechanisms, such as CRISPR-Cas and restriction-modification (R-M) systems, can further limit phage efficacy [9, 10]. Additionally, host-range limitations necessitate customized phage cocktails. Overcoming these challenges with advancement in genetic engineering, and genomics offers solutions by optimizing host specificity and enhancing phage efficacy [11, 12]. This will be crucial in establishing phage therapy as a viable strategy against AMR.

Phage characterization methods, including plaque assays (DLA) and transmission electron microscopy (TEM), have played a vital role in characterizing phage morphology, infectivity, and lifecycle. Conversely, techniques such as NanoSight, flow cytometry, and PCR-based methods are useful for counting viral particles but offer little information on their biological properties. Nevertheless, all of them skew towards phages that grow well in laboratory conditions and may miss ecologically significant but less culturable phage populations [5, 6]. As a result, the association of phages with the natural microbiome remains poorly characterized. These enumeration methods are inadequate to provide information about the genetic makeup, mutations, or viral diversity, especially in light of the SARS-CoV-2 genomic surveillance success globally. For instance, they overlook critical components like virulence factors, phage-metabolic genes and antibiotic resistance genes (ARGs), which are increasingly recognized as central to phage-microbe-host dynamics. Thus, it is necessary to uncover advanced sequencing and bioinformatics innovations to gain a comprehensive understanding of the phage community. Unlike traditional sequencing, next-generation sequencing (NGS) can process millions of fragments in parallel, allowing for a high-throughput, cost-effective, and comprehensive genome-wide analysis [13].

NGS advancements have been fundamental in enabling landmark projects such as the Human Microbiome Project (HMP) and the Metagenomics of the Human Intestinal Tract (MetaHIT) [14–16]. These initiatives applied metagenomics to characterize microbial communities and investigate the links between dysbiosis and disease progression [16]. While HMP provided microbial profiles for different body habitats, MetaHIT offered deeper insights into gut microbial interactions, including the identification of three distinct enterotypes dominated by *Bacteroides*, *Prevotella*, and *Ruminococcus* species—groups that play central roles in host metabolism and immune modulation [17]. The progress of microbiome research was significantly propelled by the utilization of NGS in both the HMP and MetaHIT projects. NGS enabled researchers to detect unculturable microbes and create more than 3000 microbial reference genomes. 16S rRNA sequencing was essential in identifying microbes at the genus and species level, while shotgun metagenomics offered valuable insights into functional gene analysis, ultimately deepening our comprehension of microbial ecosystems. Importantly, these foundational efforts also paved the way for characterizing the human phageome, which plays a key role in microbial dynamics, resistance gene transfer, and potential therapeutic applications. The metagenomic strategies developed in HMP and MetaHIT are now central to studying bacteriophage diversity, host specificity, and their influence on the AMR through transduction.

This article focuses on understanding bacteriophages from the genomic context and their role in combating AMR. The study aims to utilize the holo-transcriptomic and genomic approaches to detect bacteriophages, AMR genes and understand the distribution of phages across various disease contexts in relation to clinical outcomes. The role of transcriptionally active microbes (TAMs) in regulating the gut microbial ecosystem necessitates the need to focus on the dynamic interaction between the phages and the TAMs, which plausibly not just influences their evolutionary dynamics, but also regulates the functional human microbiome [18]. By leveraging genomic and holo-transcriptomic approaches, we can gain a deeper comprehension of disease-specific microbial interactions and phage-host dynamics.

### Detection through transcriptomic approaches

Holo-transcriptomics approach to capture the entire transcriptome gives us a thorough characterization of the host RNA and the microbial RNA within the samples. Depletion of host RNA allows for the identification of bacteriophages and opportunistic microbial entities involved in the interaction. This interaction in the host machinery is responsible for inducing the disease severity [19]. The identification of transcriptionally active microbial diversity, novel viral transcripts, and the early dynamics of host-pathogen and phage interactions can unveil significant alterations in the transcriptional activity of viral-bacterial species interplay. This approach not only enables the discovery of new phage genes and their functions but also supports functional enrichment analyses that allow for a deeper understanding of the complex interplay of phages and host relationships in the infectious cell states [20–22].

Advancements in meta-transcriptomics have significantly enhanced the characterization of recently identified RNA phages, expanding our understanding of their diversity and taxonomic classification. High-throughput sequencing has enabled the analysis of transcriptomes from tens of thousands of phages, a dramatic increase from the handful known a decade ago, with many single-stranded and double-stranded viruses, such as Cystoviruses and Leviviruses, assigned to phyla like Lenarviricota, Pisuviricota, and Duplornaviricota, by the International Committee on Taxonomy of Viruses [23].

Specialized databases complement meta-transcriptomic approaches by providing resources for annotating and analyzing the transcriptomes of characterized RNA phages. PhageScope has 873 718 partial and complete phage genomes consisting of single-stranded and double-stranded DNA and RNA viruses, host range, and taxonomy classification [24]. Within that, a subset, IMG/VR db, including RNA phage sequences from the Orthornavirae domain and other sequences with phenotype, and the Microbe *Versus* Phage database provides phage-host interactions [25, 26]. PhANNs and PhaGAA are phage annotation web servers used to annotate the presence of the phage in the data [27, 28].

Computational algorithms for phage identification can broadly be divided into two major categories. The first involves reference-based approaches, which leverage large, well-annotated phage genome databases for sequence similarity searches. These methods use sensitive alignment algorithms such as minimap2 or BWA-MEM to map sequencing reads or assembled contigs to known phage genomes, allowing for rapid and highly specific identification of previously characterized phages. The second category is *de novo* identification methods, which do not depend on existing references but instead detect putative viral sequences directly from the data. These often involve assembling quality-filtered reads into contigs or assembled genomes (MAGs), followed by annotation and classification using intrinsic genomic features and their functional meta-transcriptomic analysis, which provides insight into active phage communities and their interactions with the host. By utilising the power of holo-transcriptomics, studies have captured the transcriptionally active virome, shedding light on the dynamic roles of phages in modulating microbial ecosystems. Moreover, linking transcriptomic data with their potential functional roles enhances the ability to classify phages based on their activity which strengthens further the sequence homology based inferences [29].

### Detection of AMR through genomic approaches

By identifying mutations, ARGs, phage susceptibility, and phage contribution to bacterial virulence, genomic sequencing offers better phage selection and offers a promising alternative to antibiotics, in the fight against superbugs. Platforms like Illumina, Oxford Nanopore Technology, and PacBio facilitate complete phage genome sequencing [30]. Illumina sequencing, ideal for identifying new phages, is less reliable for phage quantification [5]. Sequence by synthesis approaches, like Illumina, is well suited for detection and assembly of previously uncharacterized phage genome sequences because of its high accuracy, high coverage and sensitivity. However, the biases introduced during library preparation and PCR amplification steps, along with short read lengths, and absence of absolute quantification without spike-in controls can distort phage abundance estimates. Moreover, single molecule high-throughput technologies such as PacBio and Nanopore support both phage enumeration and detection [31].

**Figure 1:**
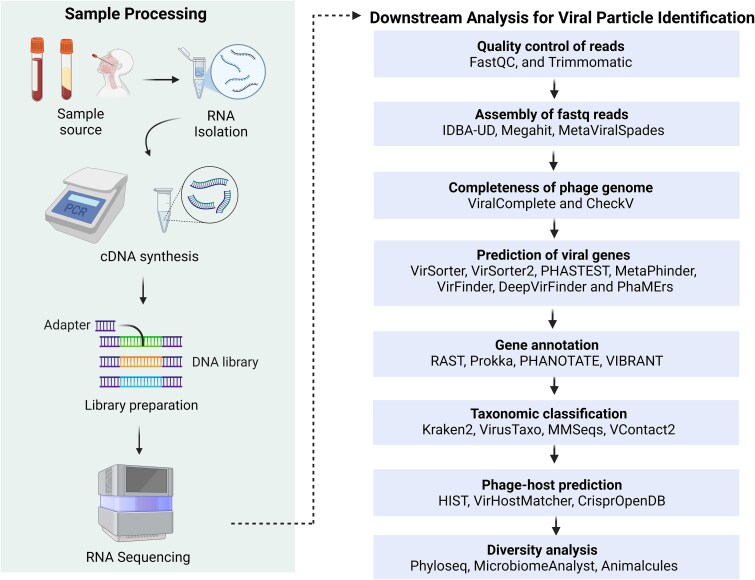
Summary of the experimental workflow and further downstream analysis. It starts with processing the sample to extract RNA, then converting it to DNA, preparing a library, and sequencing it. The data is then analyzed through various steps like checking quality, assembling the sequences, identifying viral genes, and predicting which host the virus infects. Different tools are used at each stage to help researchers better understand and classify the viruses. The figure was created using the licensed version of BioRender (https://www.biorender.com/).

Developing a sequencing protocol for bacteriophages involves sample acquisition and storage, viral particle separation, extraction of viral nucleic acids, and library preparation [32]. After library preparation and sequencing, *in silico* analysis for phage discovery streamlines the identification of phage candidates through a cohesive multi-step workflow. Raw sequencing data (.bcl files) are converted to FASTQ format using bcl2fastq. The quality of reads is assessed using FastQC [33]. To prepare high-quality reads, adapter trimming and removal of low-quality bases >Q30 and removing reads with length less than 35–50 bp [34] are performed using Cutadapt [35] or Trimmomatic [36]. Host reads are then filtered out using alignment tools like bwa-mem or Bowtie2 [37], isolating viral sequences for further analysis. These sequences undergo *de novo* assembly with metagenomic assemblers like MetaViralSpades [38], optimized with k-mer sizes (21, 31, 55 bp) for viral contig reconstruction, or alternative assemblers like MEGAHIT [39] and IDBA-UD [40, 41]. The quality of assembled contigs is evaluated using MetaQuast [42], while tools like ViralComplete and CheckV [43] assess their integrity. A significant challenge lies in distinguishing phage genomes from the bacterial regions, particularly for novel viruses lacking known homologs. To address this, tools like VirSorter2 [44], VIBRANT [45], and MARVEL [46] employ marker genes and machine learning, complemented by CNN-based DeepVirFinder and k-mer-based VirFinder [47] to enhance detection sensitivity [48]. Gene prediction, tailored to phage-specific features such as short genes and overlapping ORFs, is conducted using Prodigal [49], followed by functional annotation with Prokka, RAST, or PHANOTATE [50]. Taxonomic classification leverages k-mer-based tools like Kraken2 [51] or viroTaxo, with VContact3 [52] refining assignments through gene-content clustering. Host prediction is facilitated by tools like HIST and iPHoP [53], which harness alignment methods and CRISPR spacer databases. Despite these advancements, detecting ultra-divergent phages and resolving fragmented assemblies in low-biomass samples remains challenging. Recent advancements like Marker-MAGu [54] streamline phage and bacterial dynamics analysis directly from FASTQ files, while PhageScope [24] integrates 12 phage databases with annotations at various taxonomic levels. However, challenges in species-level annotation persist, requiring targeted solutions to improve accuracy and specificity. A wide array of bioinformatics tools supports metagenomic analysis and subsequent viral particle identification for each analytical step, as mentioned in Fig. 1 [32, 55]. Specialized tools streamline the identification and characterisation of viral contigs through virus-specific programs like Viral Informatics Resource for Metagenomic Exploration (VIROME) and Metavir2. Prophage identification tools like PhiSpy, Phage_Finder, and Prophage Finder detect distinct features like attachment sites (attP and attB) and compare the novel sequences against phage genes and proteins in the databases, enabling the discovery and characterization of new phages. These state-of-the-art genomic approaches shape the future of phage research. However, the above pipeline will evolve with renewed focus on understanding the phages, not only just in terms of their identification but also for their future therapeutic potential.

In the first-ever human case of phage therapy against the life-threatening *Acinetobacter baumannii,* bacteria developed resistance to phage cocktails (ɸPC and ɸIV) used for treatment. Whole genome sequencing (WGS) of bacterial strains before and after phage therapy revealed *A. baumannii* acquired a six base pair deletion in the glycosyltransferase gene during the course of therapy. This mutation altered capsule biosynthesis, making it difficult for the phages to infect the bacteria. Additionally, mutations in receptor genes on the bacterial surface further contributed to phage resistance [56]. A similar study on 230 *A. baumannii* strains explored the dynamics of phage susceptibility and antibiotic resistance, predicting multiple ARGs through genome sequencing. These included *sul1, sul2, tet(B), blaADC, blaOXA, blaTEM, blaCARB, mph(E), msr(E), catB8, aac(6′)-lb3, aadA1, ant(2″)-la, aph(3″)-lb,* and *armA.* Additionally, all strains carried biofilm-generating genes like *ampA, adeRS, csuE, bap, and bfmS,* contributing to their persistence and drug resistance [57]. Bacteriophages were also investigated for their potential against carbapenem-resistant *Klebsiella pneumoniae* biofilms. The genome of A¥L and A¥M phage revealed lytic genes encoding holins and endolysins, essential for bacterial cell lysis. These phages reduced the biofilm layer by 60%–70% and were free of AMR or virulence genes, making them safe for phage therapy. Notably, A¥M lacked lysogenic genes, ensuring that its viral genetic material does not integrate with bacterial DNA, further reinforcing its safety profile [58].

A bacteriophage-plasmid hybrid was identified in *Salmonella typhi*. Nanopore sequencing identified the *blaCTX-M-15* gene, carried on the P1-like bacteriophage-plasmid ph 681355 (98 kb), responsible for resistance to third-generation cephalosporins. As a part of a mobile genetic element, the *blaCTX-M-15* gene can transfer between bacterial strains, spreading resistance [59]. Another WGS study of phage-plasmids identified 184 ARGs across 1416 phage-plasmids, conferring resistance to carbapenems, aminoglycosides, colistin and fluoroquinolones. These ARGs were concentrated in specific hotspots containing integrons and transposons, genetic elements known to assist horizontal transfer of resistance genes [60].

### Distribution and dynamics of bacteriophages in a disease-specific scenario

Bacteriophages offer a natural and targeted solution against antibiotic-resistant microbes. The dynamic interaction between bacteriophages and antibiotic-resistant pathogens, mainly bacteria, sheds light on their therapeutic potential. Additionally, it explains several ways to treat both localized and systemic illnesses, showing how adaptable bacteriophage therapy is, in a range of clinical contexts and how it could be a promising adjunct or substitute for antibiotics [61].

Multidrug-resistant (MDR), pan-resistant (PDR), and extensively drug-resistant (XDR) microbes (bacteria) have emerged due to the overuse, misuse, and abuse of antibiotics. Among these, *Enterococcus faecium, Staphylococcus aureus, K. pneumoniae, A. baumannii, Pseudomonas aeruginosa,* and *Enterobacter spp.* (ESKAPE) pathogens pose a serious threat to global human health. Despite ~60 new antimicrobial drugs currently in development, their impact on combating AMR remains limited. Few of these drugs specifically target critical-priority gram-negative bacteria, including the ‘ESKAPE’ pathogens, which cause severe nosocomial and infectious diseases. Additionally, most of these drugs remain in preclinical stages, facing significant regulatory hurdles before clinical application. Meanwhile, AMR infections continue to surge, exemplified by the increasing prevalence of carbapenem-resistant *K. pneumoniae,* which is associated with alarmingly high mortality rates of 40–50%, underscoring the depletion of viable antibiotic options [62].

A comprehensive clinical report on phage therapy was published in 2012, detailing findings from the Phage Therapy Unit in Wrocław, Poland. The study included 157 patients suffering from antibiotic-resistant infections such as otitis media, deep tissue infections, osteomyelitis, and chronic bacterial prostatitis. Phage preparations were administered targeting a range of bacterial pathogens, including *Salmonella*, *Proteus*, *Escherichia coli*, *Pseudomonas*, *Staphylococcus*, *Enterococcus*, and *Klebsiella* species. The analysis revealed that 39.9% of patients (n = 61) exhibited a favorable therapeutic response, while 60.1% (n = 92) experienced insufficient or no clinical improvement, highlighting the ongoing challenges in achieving consistent efficacy with phage therapy [63].

The advancement of anti-*Staphylococcus* phage cocktails has demonstrated that the majority of common strains can be successfully combated with a mix of just six phages. The abundance of virulent phages targeting *S. aureus*, such as φ812, which infects hundreds of strains, highlights the immense potential of phage therapy [64]. Mice treated with oral phage treatment for gut-derived *P. aeruginosa* sepsis had a 66.7% survival rate. Additionally, a single intraperitoneal phage strain against vancomycin-resistant *E. faecium*, β-lactamase-producing *E. coli*, and imipenem-resistant *P. aeruginosa* produced 100% survival in bacteraemia models, indicating the potential use of bacteriophages as a life-saving substitute against resistant infections [63, 65]. More recently, phages have been successfully used in the treatment of patients with Methicillin-resistant *S. aureus* (MRSA) infections that would otherwise have required amputation, where Sb-1 phage played a crucial role. Further reports demonstrate the use of phages against other resistant pathogens, such as *E. coli*, *Burkholderia cepacia*, and *A. baumannii* [64]. Ongoing clinical trials in Europe are also investigating the efficacy of phages against infections caused by resistant *P. aeruginosa* [64]. Li Huang *et al.* reported 22 types of *A. baumannii* bacteriophages. Abp95 was found to be a potential option because of its broad host range, which allowed it to efficiently target 29% of *A. baumannii* strains. Notably, Abp95 showed great therapeutic promise by successfully eliminating MDR *A. baumannii* infections and speeding wound healing in a diabetic mice infection model [66].

The dynamics of bacteriophages in microbial diseases are characterized by rapid coevolutionary processes, including lytic and lysogenic cycles, and their impact on bacterial populations. Lytic phages, which infect and lyse their bacterial hosts, offer therapeutic potential, as demonstrated in a recent study where they have effectively targeted *E. coli* or *K. pneumoniae* in clinical settings, reducing bacterial burden and restoring microbial balance [67]. A 2025 study published in the *Journal of Clinical Investigation* highlighted that phage predation alters bacterial population dynamics by selectively eliminating susceptible strains, which can, however, promote the emergence of phage-resistant mutants [68]. Conversely, temperate phages, which can integrate into the bacterial genome as prophages, contribute to lysogeny and long-term bacterial adaptation. In natural microbial communities, lysogeny is prevalent, yet its dynamics in disease contexts remain underexplored. A study found that temperate phages exhibit low activity under high bacterial density but can switch to lytic cycles under stress conditions, such as antibiotic exposure, worsening dysbiosis in diseases like inflammatory bowel disease.

Phage-bacteria coevolution also drives microbial community dynamics in disease. Phage predation pressure leads to rapid evolutionary changes in bacterial populations, such as mutations in surface receptors, which in turn influence phage infectivity. In a cholera model, phage-bacteria coevolution was shown to suppress disease severity by reducing *Vibrio cholerae* populations but simultaneously promote antibiotic resistance, highlighting a complex trade-off in phage dynamics [69]. These dynamics offer insights into microbial disease progression and highlight the therapeutic potential of phages, provided their evolutionary impacts are carefully managed.

**Figure 2:**
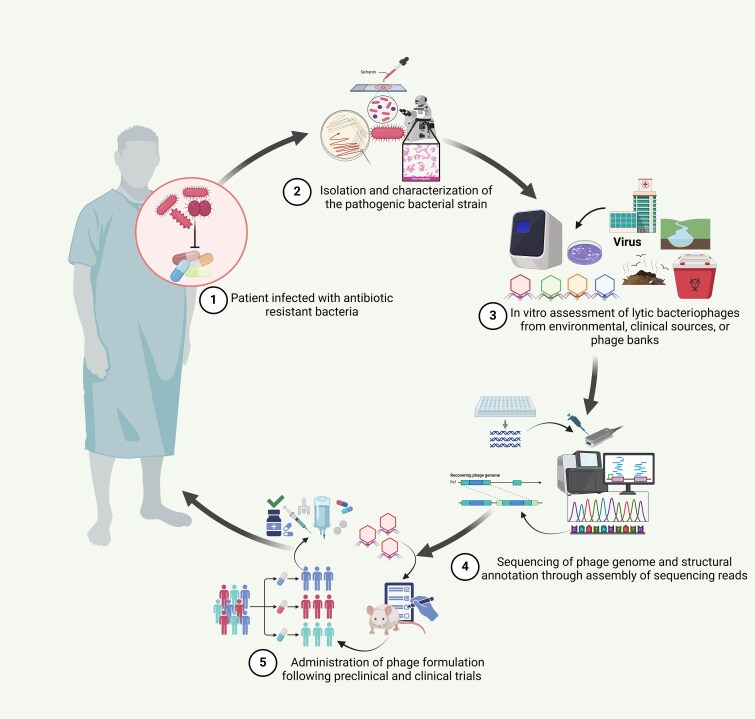
Overview of the phage therapy steps, from screening and selecting specific bacteriophages through sequencing to clinical trials for treating AMR bacterial infections. This schematic outlines the key steps in phage therapy against MDR bacterial infections: (1) identification of antibiotic-resistant infection, (2) isolation of the pathogen, (3) *in vitro* screening of lytic phages from various sources, (4) genomic validation for safety and specificity, and (5) development and clinical evaluation of phage formulations for therapeutic use. The figure was created using the licensed version of BioRender (https://www.biorender.com/).

Figure 2 illustrates the steps of phage therapy, from identifying resistant pathogens, screening specific lytic phages, validating their safety and specificity through genomic analysis, and formulating them for clinical use. This strategy presents a promising solution to combat AMR.

During the COVID-19 pandemic, phage therapy emerged as a potential adjunct treatment, particularly for managing secondary bacterial infections in severely ill patients. A notable double-blind clinical trial in Iran assessed the use of inhaled phage therapy in 60 hospitalized COVID-19 patients with moderate to severe illness. Patients receiving phage therapy alongside standard care showed significant improvements in oxygen saturation, reduced fever and dyspnea, shorter hospital stays, and lower incidence of secondary bacterial pneumonia compared to the control group receiving standard care alone [70]. Further, a study characterized a novel phage, vB_KshKPC-M, specifically targeting carbapenemase-producing *K. pneumoniae* strains isolated from VAP cases in COVID-19 patients. Genomic analysis confirmed its therapeutic potential, reinforcing the role of phage therapy as a valuable tool in combating resistant secondary infections during pandemics [71]. Another study revealed how phage therapy successfully controlled the *A. baumanii* infections in COVID-19 patients in China. A 2-phage cocktail administered to four critically ill patients reduced the bacterial burden in all cases, highlighting the potential of phage therapy in managing secondary infection outbreaks in COVID-19 settings [72]. In 2020, the phage-based company APT received emergency FDA approval for phage therapy trials targeting secondary bacterial infections in COVID-19 cases, especially *A. baumanii* and *S. aureus* (NCT04636554) [73].

### Understanding disease severity and clinical outcome during phage therapy

Bacteriophages comprise the major portion of the host virome and lead to a constant trade-off between bacteria and phage [74]. Treating infectious diseases spurred on by bacteria resistant to antibiotics has shown notable effectiveness. The high specificity of phages enhances their clinical application [75].

A study investigated different sequencing technologies to understand how bacteriophages contribute to bacterial virulence and AMR genes in three different Shiga-toxin producing *E. coli*. MiSeq generated small fragments failed to detect the entire phage genome, however long read sequencing using MinION Nanopore and PacBio allowed more accurate analysis and detected the location of phage insertion [76]. Results reveal variation in the location of the Stx1 phage gene in the bacterial strain. The bacterial strains CFSAN027343, CFSAN027346, and CFSAN027350 had the Stx1 gene at the torS-torT intergenic region, the classic wrbA site and the mlaA-ypdK intergenic site, respectively. This variation influences the severity of the infection and bacterial virulence among strains [76]. Additionally, the strain CFSAN027346 was recorded to have an extra plasmid carrying multiple AMR genes, including dfrA, aph(3″)-Ib, aph(6)-Id, tetB, sul2, and blaTEM-1B. The study confirms long-read sequencing as a reliable and cost-effective tool for genome closure and detecting virulence markers. Particularly, the identification of AMR gene loci within the phage genomes provide critical insights into the mechanisms driving the dissemination of resistance traits. By mapping these genes, researchers can trace patterns of resistance spread across the bacterial populations, assess the potential for horizontal gene transfer mediated by phages, and develop informed strategies for public health surveillance, outbreak tracking, and containment of AMR [76].

Intravenous administration of phage cocktail of three anti–*S. Aureus* phages cleared the infection in a patient after repeated failure of antibiotics. 16S rRNA gene sequencing revealed that phage therapy did not disrupt the gut and salivary microbiome, although the skin microbiome fluctuated slightly. Shotgun sequencing detected the presence of ARGs for tetracyclines, macrolides, and β-lactams before therapy, while no new resistance was found after the therapy. Unlike conventional antibiotics that often cause broad disruptions to the host microbiome, this case study highlights the microbiome-sparing potential of phage therapy. Moreover, a study demonstrated minimal impact on the patient’s microbiome following phage therapy targeting *S. aureus,* emphasising the need for clinical trials to further investigate how phage therapy affects the microbiome. This finding stands out significantly since conventional antibiotics are often linked to gut microbiome disruption [77]. WGS of *K. pneumoniae* in primary sclerosing cholangitis revealed a high abundance of *K. pneumoniae* and the presence of ARGs. Shotgun metagenomics and 16S rRNA analysis predicted a drop in Kp levels following treatment. The microbiome diversity was undisturbed, and no new resistance genes emerged [78].

### Presence of drug-resistant TAMs and their associated bacteriophages

TAMs are essential for microbial ecosystems, as they represent gene expression in response to environmental conditions. These microbes indicate their presence for maintaining the overall function and stability of microbiomes [19]. Some bacteriophages have been observed to regulate bacterial transcription factors, effectively controlling host metabolism [79].

Bacteriophages might affect TAMs through various mechanisms, including lysogenic conversion and lytic infections, both of which can impact microbial gene expression significantly. In the lysogenic phase, phages incorporate themselves into the bacterial genome, modifying transcriptional activity by controlling host genes, which can occasionally provide benefits like antibiotic resistance or increased virulence [80]. In contrast, lytic phages take over the transcriptional processes of bacteria to replicate, resulting in metabolic changes within their hosts [81]. Some bacteriophages have been observed to regulate bacterial transcription factors, effectively controlling host metabolism and even enhancing bacterial survival under specific environmental conditions [79]. This dual influence of phages on microbes highlights the complex evolutionary dynamics between the viruses and their microbial hosts, specifically TAMs. The use of phage in therapy depends upon the understanding of TAMs to predict bacterial responses to infection and optimize therapeutic efficacy [80]. Furthermore, scientists are studying engineered bacteriophages that can alter the transcriptional activity of bacteria for synthetic biological purposes, such as microbiome manipulation and specific regulation of bacterial genes [82].

### AMR bacteria beyond ESKAPE pathogens

Beyond the well-known ESKAPE pathogens, which are resistant to multiple classes of antibiotics, there exists a group of other pathogens that also pose significant challenges in clinical conditions. These pathogens are not only resistant to conventional antimicrobial treatments but are also actively transcriptionally involved, as they express a wide array of genes that contribute to their adaptability and survival in hostile environments.

According to previous studies from our lab, it has been identified that in addition to the ESKAPE group, there are other pathogens which are transcriptionally active and play a major role in differential disease severity. We identified a list of 80 pathogens from hospital-admitted SARS-CoV-2-infected patients and 121 pathogens from the Dengue patients [18, 19, 21]. Upon reviewing literature and the BV-BRC database, we found that 23 out of the 80 COVID-19-related pathogens had AMR phenotype. Similarly, 22 out of the 121 dengue-related pathogens showed AMR phenotype [83]. Further analysis revealed the presence of 19 microbial species in COVID-19 and dengue data each, having phages that can target them (Supplementary Tables 1 & 2). The total presence of 5735 phages (for both SARS-CoV-2 data and Dengue data) targeting these AMR pathogens opens up exciting possibilities for developing phage-based treatments specific to bacterial species. Phages infecting these transcriptionally active, AMR-associated pathogens may exert therapeutic effects either through direct lysis or by perturbing bacterial transcriptional networks crucial for survival under host stress. For instance, *E. coli*, the most phage-rich species in the dataset, is not only a common gut colonizer but also a major cause of extraintestinal infections with high resistance potential [84]. Similarly, pathogens like *Pasteurella multocida* [85, 86] and *Streptococcus suis* [18, 87–89], though less frequently discussed in mainstream AMR discourse, displayed substantial phage presence, highlighting their ecological and clinical relevance. The interactions between these TAMs such as *Vibrio spp.*, also display intricate phage-host dynamics but remain comparatively underexplored [90]. *Vibrio spp.*, though globally relevant in cholera and wound infections, remain underrepresented in transcriptional and phage interaction studies, despite emerging evidence of their adaptive resistance mechanisms [90, 91]. The identification of disease-specific microbes, such as *V. cholerae* in dengue and *Mycobacterium tuberculosis* in COVID-19, further underscores the need to expand our AMR surveillance beyond ESKAPE pathogens.

### Correlation of host-microbiome and bacteriophages

The ubiquitous tendency of microorganisms makes them prevalent in various areas that include living organisms. Therefore, it is imperative to evaluate the interactions between the microorganisms and their shared hosts. The microbes in the human gastrointestinal (GI) tract have unique characteristics that provide different benefits to the human hosts, including proper digestion and metabolism, production of essential vitamins, enabling gut barrier protection, and maintaining a healthy immune system [92, 93]. The gut microbiome is composed of both pathogenic and non-pathogenic microbes and can be impacted by several factors; however, the population of pathogenic bacteria cannot be fully eradicated from the system. A healthy symbiosis between the pathogenic and non-pathogenic microbial communities underlies overall health status [94]. Cumulative data from various studies have shown that dysbiosis of healthy gut microbiota can hamper essential physiological processes such as insulin sensitivity, functioning of the nervous system, GI activity, and emotional regulation [95, 96].

Akin to bacteria, bacteriophages are abundant life forms that are ubiquitously present even inside the human body. The ratio of virotypes with respect to species-level bacterial population is about 10:1 [97]. This implies that there is a vast viral population that is still unexplored inside the gut microbiome, which can aid in providing a better understanding of human gut populations. Studies that employed metagenomic analysis on the viral population obtained from fecal matter have revealed that ~81%–93% of the bacterial viruses in the GI tract are novel at that point of time and cannot be traced to a definitive taxonomic position of the compatible bacterial host [98]. Research in healthy human hosts has also brought forth data suggesting that a significant diversity exists between the phageomes in different individuals. Minot *et al.* delineated that gut bacteriophages exhibiting a lysogenic life cycle and remaining in the human body for an extended time, with their respective bacterial host had a slower rate of evolution than the lytic phages in the system. Additionally, these gut bacteriophages interact with specific strains of bacteria, thereby keeping the ratio of phage to bacteria at 1:1 [99].

### Mutualistic interaction between bacteriophages and gut bacteria

**Figure 3:**
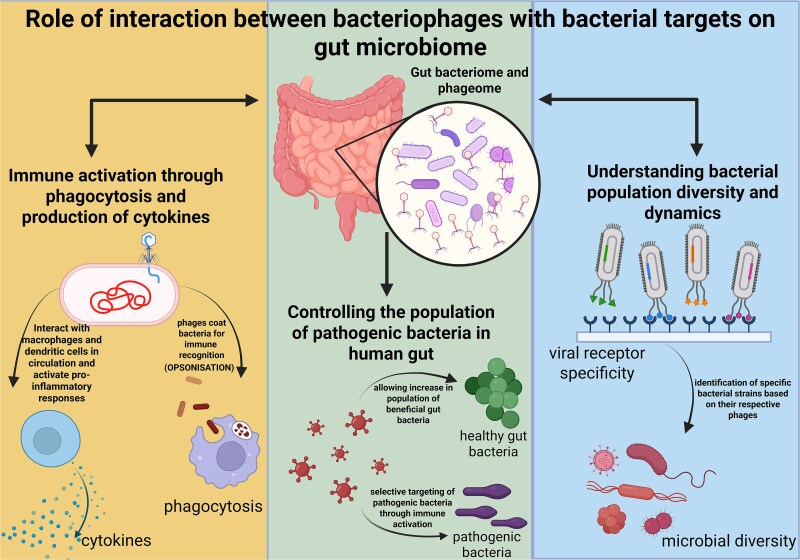
The effect of the interaction between a bacteriophage and its specific bacterial target on the human host. The phage population present in the human gut (phageome) helps to control the population of harmful bacteria by selectively targeting them and allowing the population of healthy bacteria to grow. Phageome elicits immune activation by internalising inside the human cell and activating the viral detection receptors and resulting in the production of inflammatory cytokines and activation of macrophages. Gut phageome can also reveal insights into the diversity in bacterial population by tracing the respective host organisms for gut bacteriophages. Bacteriophages also tend to coat bacteria for easier recognition by the immune system (opsonization), thereby enabling them to get phagocytosed. The figure was created using the licensed version of BioRender (https://www.biorender.com/).

A complex relationship exists between a bacteriophage and its respective bacterium inside the gut environment. Phages and bacteria can exhibit either an antagonistic relation or mutualistic relation with each other; for instance, a phage can lethally attack a bacterium during a lytic cycle, or it can take shelter inside the bacterial cell for a longer duration and derive specific benefits from its bacterial host. These relations evolve over time as phage and bacteria are predisposed to high evolutionary tendencies. These interactions, therefore, can greatly influence the gut microbiota composition [100]. The plasticity and diversity of the bacteriophage genome bestow them with great adaptability to the GI tract and form niches inside the GI tract. Studies conducted on gut-on-a-chip mucosa found that phage populations evolved especially concerning their capsid protein, leading to a modified glycan-binding phenotype giving them an evolutionary advantage over their ancestral phages. These phages with altered capsid protein, Hoc, promoted better adherence to the mucus membrane through human fucosylated mucin glycan and got further localized towards the bacterial hosts. Along with this, increased variations in the phages with unique mutational profiles were also discovered in mammalian mucosal environments [101]. Based on these studies, it can be concluded that bacteriophages regulate the bacterial density in the gut microbiome and also affect their diversity by restricting bacterial virulence genes by dysregulating their host’s defense systems. Bacteriophages have been known to impact the human immune system indirectly; however, recent evidence has shed light on the fact that they do influence human immunity directly. This takes place through the process of phagocytosis and cytokine release as shown in Fig. 3, thereby modulating innate as well as adaptive immunity. The phage interaction with commensal bacteria alters the microbial equilibrium and thereby impacts the immune system and aids the spread of pathogenic bacteria [102].

### Challenges of phage therapy

Phage therapy, which uses lytic bacteriophages to infect and destroy pathogenic bacteria, offers a promising alternative to traditional antibiotics, particularly in an era of mounting AMR. Lytic phages hijack bacterial cellular machinery to replicate, ultimately lysing the host. However, the effectiveness of this approach is challenged by three major factors. First, bacteria have evolved complex defence mechanisms, including CRISPR-Cas systems, R-M pathways, and abortive infection programs, that can recognize and neutralize invading phage DNA. Second, the inherent genomic heterogeneity of phages, driven by high mutation rates and HGT, complicates their classification, and therapeutic standardisation. Third, ethical and regulatory hurdles, such as the absence of harmonized clinical guidelines, difficulties in patenting natural phages, and personalized trial designs, continue to obstruct the clinical integration of phage therapy. Together, these factors underscore the need for multidisciplinary strategies to unlock the full therapeutic potential of bacteriophages.

### Mechanisms of microbial defence against bacteriophages

The CRISPR-Cas system is a vital microbial defence mechanism where Cas9 and Cas12a endonucleases confer bacterial immunity by cutting phage DNA with target-strand nicking adequate to suppress phage replication [9]. CRISPR specificity and mismatch tolerance are crucial in shaping phage evasion strategies. R-M systems include a modification enzyme that adds a methyl group to specific DNA sequences, and a restriction enzyme that cuts DNA that does not have this protective methylation [10]. Abortive infection (Abi) systems function as a bacterial defence strategy that disrupts phage replication by inducing programmed cell death in infected bacteria, thereby preventing the release of new phage particles. In *E. coli*, the AbpAB system detects single-stranded DNA-binding proteins (Gp32) from phages, activating Abi and inhibiting phage replication [103]. A helicase/nuclease-based Abi system in *Sinorhizobium meliloti* degrades viral DNA and triggers bacterial cell death. The *hna* gene, widely distributed across bacteria, initiates Abi response upon detecting phage-encoded single-stranded DNA-binding proteins, ensuring infected cells die without releasing progeny [104]. The Bacteriophage Exclusion (BREX) system in bacteria employs DNA methylation strategies against phages. Unlike the R-M system, BREX does not degrade foreign DNA; instead, it modifies the bacterial genome to distinguish self from non-self, thereby blocking phage replication. In *Bacillus cereus*, the BREX system obstructs phage DNA replication, thereby conferring resistance to a range of phages [105]. These systems working together produce a multi-layered defensive system against bacteriophage infections.

### Phage genomic heterogeneity

Phage genomic diversity arises due to high mutation rates, genetic mosaicism, and HGT, resulting in significant variability within the phage genomes [8]. This results in unpredictable host interactions and makes the classification and standardisation of therapeutic phages more complex. *Pseudomonas* phage MD8 undergoes extensive gene exchange and structural variation [106]. Phages facilitate HGT through transduction, allowing the transfer of genetic material, including antibiotic-resistance genes and virulence factors, between the bacterial hosts, which can complicate therapeutic applications [107]. During phage replication, the pac site acts as the initiation point for DNA packaging, where the packaging machinery recognizes and cleaves the DNA for encapsulation into new phage particles. Similarly, the cos site, a cohesive end sequence, is identified by terminase enzymes, ensuring precise genome cutting and packaging [108]. These packaging strategies—pac-type and cos**-**type, contribute to phage heterogeneity, influencing genome organization, host range, and mechanisms of gene transfer. While HGT promotes genetic diversity, it complicates phage therapy by spreading resistance genes, potentially leading to MDR bacteria [7]. To mitigate these risks, several biosafety strategies are critical for effective and responsible phage therapy. First, strict selection of lytic phages over lysogenic phages is essential, as lytic phages are less likely to mediate ARG transfer [109]. Second, screening phages for the absence of ARGs is imperative to avoid therapeutic agents that may inadvertently promote resistance. Cases such as *S. aureus* phages transferring resistance islands highlight this concern [110, 111]. Third, it is advisable to avoid phages that demonstrate lysogeny or harbour prophage genes, even if they appear lytic under some conditions, due to the risk of induction and subsequent gene transfer [112]. Lastly, contextual factors within the host environment (*e.g.* microbial density, immune pressures) can influence whether phages act in a therapeutic *versus* transductive capacity, making environment-aware design of phage therapy increasingly necessary [113].

Research indicates that phages frequently undergo mutations, particularly in genes encoding receptor-binding proteins such as tail fibres or tail tubular proteins, which are critical for recognising and binding to bacterial hosts. These mutations can enhance the phage’s ability to infect bacterial hosts. A study identified a spontaneous mutation in the gene of *Acinetobacter* phage vB_Ab4_Hep4 encoding tail tubular protein B, expanding the phage’s host range by altering its receptor binding properties [114]. Another study on T3 phages demonstrated modifying host-range determining regions in tail fibre proteins through mutagenesis that suppresses bacterial resistance and enhances phage adaptability [115].

### Regulatory and translational challenges in phage therapy

Despite the therapeutic promise of phage therapy in combating AMR its integration into mainstream clinical practice remains obstructed by multiple regulatory, ethical, and translational challenges. Many researchers believe that phage therapy’s progress has been slowed by challenges in securing intellectual property rights. Since phages occur naturally, patenting them is difficult, which in turn discourages pharmaceutical investment and commercial interest [116]. Another regulatory hurdle is the need for clinical trials to prove phage safety and efficacy. Before trials, production and distribution must be considered. Government bodies like the National Institute of Health could create phage libraries, enabling approved labs, hospitals, and doctors to access them. Universities may also host libraries, but stable, large organisations are likely needed to ensure global, uninterrupted access despite political instability [117] Compounding to this, is the mismatch between regulatory expectations for chemical drugs *versus* biologics like phages, which often require personalized formulation and adaptation to specific pathogens. This has led to the argument that traditional regulatory models are ill-suited for live biological agents, as highlighted in recent evaluations of global phage programs [118]. From an ethical standpoint, the reliance on compassionate use protocols for patients with drug-resistant infections, in the absence of approved alternatives, raises concern about informed consent, safety assurance, and equitable access [119]. Additionally, clinical trial design remains a fundamental bottleneck. Many trials have failed not because of phage inefficacy but due to poor matching between phage cocktails and infecting bacterial strains, suboptimal phage titers, and lack of adaptive trial models. Phages require rapid personalisation, which current randomized control trial frameworks are not designed to accommodate [119, 120]. Furthermore, ensuring Good Manufacturing Practices (GMP), safety, and product consistency in phage production adds another layer of complexity. Without international consensus on quality control, potency testing, and batch reproducibility, translational progress remains slow. Recent efforts have emphasized the need for phage banks, adaptive regulatory policies, and improved preclinical characterisation protocols to overcome these hurdles [121]. Collectively, these challenges underscore that advancing phage therapy from the lab to the clinic requires coordinated efforts in policy reform, ethical oversight, and innovative trial methodologies.

### Opportunities in phage therapy

With advances in high-throughput sequencing and powerful genomic tools, bacteriophage research is rapidly evolving, enabling precise genomic mapping and the identification of key evolving elements such as tail fiber proteins and receptor-binding domains across different global regions [34, 122]. Tools like SpikeHunter leverage deep learning to predict phage-host interactions, aiding targeted therapy against pathogens like *Acinetobacter* and *E. coli* [123].

Phage cocktails, combinations of phages targeting multiple bacterial strains, are emerging as a powerful tool against antibiotic-resistant infections. Genomic surveillance of *A. baumannii*, using quality trimming, MLST typing, and AMR profiling, has revealed region-specific strain prevalence, guiding the design of targeted phage mixtures [124]. By integrating genomic data from both the phages and their bacterial hosts, such as sequence types, virulence factors, and resistance markers, researchers-clinicians’ partnership can develop cocktails that cover diverse bacterial genotypes while reducing cross-resistance risks [125]. A metagenomic comparison of phage cocktails from 1997 and 2014 showed significant compositional shifts, identified through sequencing, which highlights the need for continuous refinement efforts in the field to combat possible evolving MDR bacteria [126].

In the fight against AMR, genetic engineering of bacteriophages has emerged as a powerful strategy to enhance their therapeutic potential. Advanced genome-editing techniques such as CRISPR allow precise modifications to phage genomes, enabling them to efficiently target and eliminate antibiotic-resistant bacteria. Strategies for altering phage genomes to enhance their effectiveness against AMR include homologous recombination and CRISPR-Cas systems [12]. Homologous recombination facilitates genetic changes through recombination between the phage genome and an introduced editing template during infection. CRISPR-Cas enables targeted removal of ARGs from the bacterial genomes, thus restoring antibiotic efficacy [127]. Furthermore, synthetic biology approaches such as Gibson assembly and transformation-associated recombination enable the construction of custom phage genomes outside the bacterial hosts, making it easier to edit the genome at any position, which are subsequently introduced into bacteria for ‘rebooting’ [128–130]. These methods enhance phage safety, effectiveness, and host range by allowing precise genetic modifications and expanding phage capabilities. Additionally, next-generation sequencing technology ensures accurate verification of genetic alterations made through CRISPR-Cas techniques [131, 132].

Another key strategy is modifying phages to expand their host range by altering receptor-binding proteins, such as tail fibres and spike proteins. For example, researchers modified *E. coli* phage T7 to infect a broader range of bacterial strains [11, 133]. A GFP-tagged *E. coli* phage PP01 was engineered to act as a biosensor and detect *E. coli* O157:H7 in wastewater [134]. Additionally, phages can be designed to carry antimicrobial genes like small acid-soluble spore proteins (SASPs) to kill antibiotic-resistant bacteria such as MRSA [135]. Phages are also being developed to target biofilms. A modified T7 phage that produces the DspB enzyme was able to eliminate up to 99.997% of *E. coli* biofilms [136]. This could help treat infections where biofilms make bacteria more resistant to antibiotics. With advances in synthetic biology, engineered phages are becoming powerful tools in the fight against AMR, offering new treatment options where traditional antibiotics fail.

**Figure 4:**
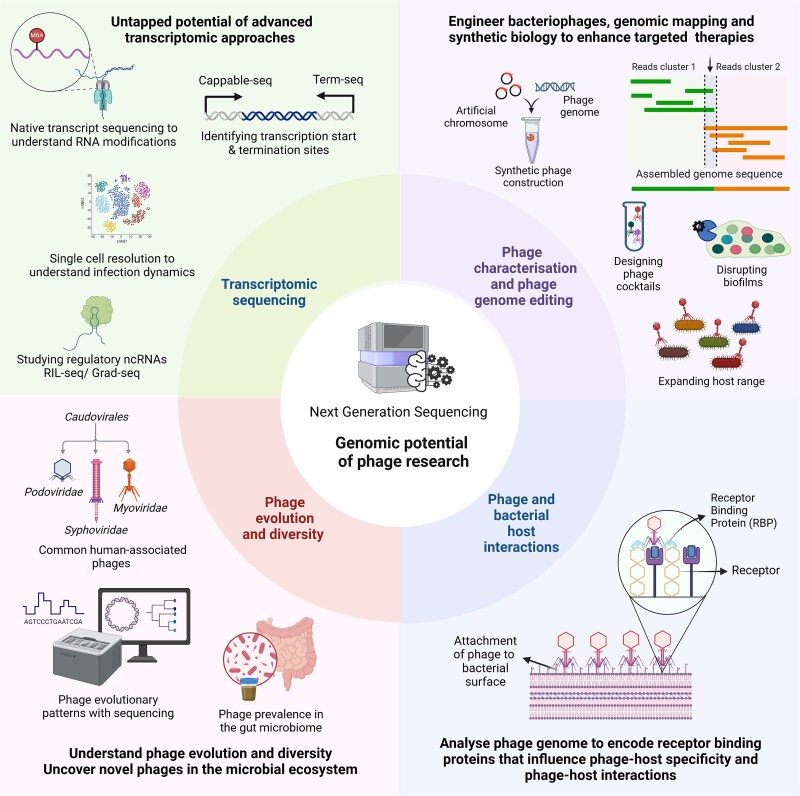
Genomic advancements and potential for future phage research. Key areas of upcoming bacteriophage research revolve around the development of phage cocktails based on genomic profiling, synthetic biology approaches for engineering phages, analysis of receptor binding domains to study phage-host interaction, phage evolutionary dynamics, and diversity in the human microbiome, and to utilize advanced transcriptomic approaches. The figure was created using the licensed version of BioRender (https://www.biorender.com/).

Differential RNA-Seq and Cappable-Seq serve as powerful transcriptomic tools, employed to map transcription initiation sites, while Term-Seq elucidates transcription termination sites [137–139]. Moreover, non-coding RNAs play a critical role in modulating gene expression through interactions with RNA-binding proteins (RBPs) as investigated using techniques such as RIL-seq, Grad-seq, and CLIP-seq [140–142]. Bacteriophages may also modify their RNA to circumvent bacterial defence mechanisms, analogous to their DNA modification strategies. Techniques such as Nanopore direct RNA sequencing and m6A-Seq facilitate the detection of modifications like m6A, providing deeper insights into survival strategies [143, 144]. Single-cell resolution enables analysis of phage-bacteria interactions, which may help to uncover bacterial survival strategies and infection heterogeneity [107]. These state-of-the-art approaches create new avenues for research in the AMR crisis, as illustrated in Fig. 4.

## Conclusion and future prospects

With the growing complexity of bacterial resistance, particularly in transcriptionally active strains and the global rise of AMR, phage therapy represents a novel and potentially effective approach as compared to traditional antibiotic treatment. In the study of bacteriophages, NGS has played a crucial role in unraveling viral genomes, mutations, the identification of ARGs, and phage susceptibility. Genomic sequencing of bacteria reveals multiple ARGs, while bacteriophage sequencing predicts the presence of lytic enzymes required for bacterial lysis and the absence of virulence genes, ensuring a safe phage therapy. Phage therapy has shown promising results in patients suffering from chronic bacterial prostatitis, MRSA infections, and osteomyelitis. Metagenomic studies reveal the dynamic interactions between bacteria and phages and highlight the major role of phages in regulating the microbiome, compared to bacteria. Phages limit pathogen overgrowth, restrict bacterial virulence, and promote commensal bacteria by building a healthy ecological niche. These regulations within humans assist in analyzing disease severity and clinical outcomes. Advancements in high-throughput sequencing in association with machine learning approaches and synthetic biology allow enhanced comprehension of the phage genome, genome editing, and receptor binding proteins for bacteria-phage interactions.

Infectious diseases like COVID-19 and dengue have been studied for the role of TAMs in disease severity, but the role of bacteriophages remains less explored. Since they are known to shape the host microbiome through bacterial interactions, future research in this domain should advance our knowledge in phage biology. WGS can aid in identifying new phage populations and reveal evolutionary relationships. The impact of phage-bacteria associations in infectious diseases remains largely unexplored. Holo-transcriptomics can be harnessed to explore the gene expression dynamics of both bacteriophage and hosts during infection, which is crucial for understanding phage replication, host defense mechanisms, and their impact on disease severity and clinical outcomes. Information on the presence of specific phages and their association with commensal and opportunistic bacteria will facilitate understanding of disease progression. Moreover, genomic data is useful in the search for appropriate phages from phage banks, enabling personalized treatment against resistant microbes. Investigating the phage genome will also highlight genes that are responsible for phage-host specificity, which can be used to target bacterial pathogens. Phage therapy has the ability to evolve into a potent and adaptable strategy to address the global AMR epidemic.

**Key Points**

- High throughput sequencing captures phage genome and detects antimicrobial resistance genes, allowing a deeper understanding of phage-mediated therapeutics.
- Phage therapy holds potential as an alternative to antibiotics, targeting multidrug-resistant superbugs, including ESKAPE pathogens.
- Analysis unveils 19 bacterial species linked to AMR in COVID-19 and Dengue data, highlighting the emerging superbugs alongside the globally known ESKAPE pathogens.
- The interaction between bacteriophages and the gut bacteriome shapes AMR and host physiology by targeting resistant bacteria which also regulates microbial diversity.
- Phage cocktail and phage genome engineering are transforming precision in phage therapy by restoring antibiotic sensitivity.

## Supplementary Material

Supplementary_Tables_elaf011

## Data Availability

There is no data to report.

## Notes

**Basudha Banerjee** is interested in expanding bacteriophage genomics and phage therapy to combat antimicrobial resistance (AMR).

**Sayanti Halder** is involved in understanding disease severity through dengue, bacteria, and human whole-genome sequencing.

**Shubham Kumar** focuses on next-generation sequencing–data analysis with a focus on single-cell data from dengue patients.

**Muskan Chaddha** is interested in understanding the role of bacteriophages in the therapeutic aspect against AMR.

**Raiyan Ali** is engaged in developing optimized workflows and pipelines for bulk and single-cell sequencing data analysis.

**Ramakant Mohite** is interested in optimizing sequencing workflows as well as investigating host genetic predisposition and viral genetics influencing disease outcomes.

**Muskan Bano** is interested in evaluating the role of bacteriophages in AMR.

**Rajesh Pandey’s** lab is working towards INtegrative GENomics of Hope-PathogEn (INGEN-HOPE) with focus on identifying hierarchical modulators of disease severity sub-phenotypes (mild, moderate, severe) and clinical outcomes (recovery and mortality).
