# Supplementary material for: Genomic insights into bacteriophages: a new frontier in AMR detection and phage therapy
Source: Brief Funct Genomics. 2025 Jul 28;24:elaf011. doi: 10.1093/bfgp/elaf011 (PMC12302716; doi:10.1093/bfgp/elaf011)
Supplement: Supplementary_Tables_elaf011 [file supplementary_tables_elaf011.docx]

**Supplementary Table 1**. Out of the 80 TAMs from COVID-19 data, we found 19 bacterial species with AMR traits and their associated phages, as per the BV-BRC database.

| **COVID-19 pathogens** | **Total Phages (Source:** [**BV-BRC**](https://www.bv-brc.org/view/GenomeList/?eq(genome_name,phage))**), accessed on 19th February, 2025** |
| --- | --- |
|  |  |
| *Acinetobacter baumannii* | 110 |
| *Burkholderia pseudomallei* | 14 |
| *Campylobacter jejuni* | 69 |
| *Clostridioides difficile* | 9 |
| *Enterobacter cloacae* | 23 |
| *Enterococcus faecalis* | 89 |
| *Escherichia coli* | 1098 |
| *Haemophilus influenzae* | 2 |
| *Klebsiella pneumoniae* | 312 |
| *Mycobacterium tuberculosis* | 6 |
| *Pasteurella multocida* | 5 |
| *Providencia stuartii* | 1 |
| *Pseudomonas aeruginosa* | 391 |
| *Salmonella enterica* | 357 |
| *Staphylococcus aureus* | 186 |
| *Staphylococcus haemolyticus* | 3 |
| *Stenotrophomonas maltophilia* | 32 |
| *Streptococcus pneumoniae* | 123 |
| *Streptococcus suis* | 83 |
| **Total** | **2913** |

**Supplementary Table 2**. Out of the 121 TAMs from dengue data, we found 19 bacterial species with AMR traits and their associated phages, as per the BV-BRC database.

| **Dengue pathogens** | **Total Phages (Source:** [**BV-BRC**](https://www.bv-brc.org/view/GenomeList/?eq(genome_name,phage))**), accessed on 19th February, 2025** |
| --- | --- |
|  |  |
| *Acinetobacter baumannii* | 110 |
| *Burkholderia pseudomallei* | 14 |
| *Campylobacter jejuni* | 69 |
| *Clostridioides difficile* | 9 |
| *Enterobacter cloacae* | 23 |
| *Enterococcus faecalis* | 89 |
| *Escherichia coli* | 1098 |
| *Haemophilus influenzae* | 2 |
| *Helicobacter pylori* | 27 |
| *Klebsiella pneumoniae* | 312 |
| *Mycobacterium tuberculosis* | 6 |
| *Pasteurella multocida* | 5 |
| *Providencia stuartii* | 1 |
| *Pseudomonas aeruginosa* | 391 |
| *Salmonella enterica* | 357 |
| *Staphylococcus aureus* | 186 |
| *Staphylococcus haemolyticus* | 3 |
| *Stenotrophomonas maltophilia* | 32 |
| *Vibrio cholerae* | 88 |
| **Total** | 2822 |
